# PLD-Specific Small-Molecule Inhibitors Decrease Tumor-Associated Macrophages and Neutrophils Infiltration in Breast Tumors and Lung and Liver Metastases

**DOI:** 10.1371/journal.pone.0166553

**Published:** 2016-11-16

**Authors:** Karen M. Henkels, Naveen Reddy Muppani, Julian Gomez-Cambronero

**Affiliations:** 1 Center for Experimental Therapeutics and Reperfusion Injury, Brigham and Women Hospital and Harvard Medical School, Boston, Massachusetts 02115, United States of America; 2 Wright State University Boonshoft School of Medicine, Department of Biochemistry and Molecular Biology, Dayton, Ohio 45435, United States of America; Queen Mary University of London, UNITED KINGDOM

## Abstract

Phospholipase D-2 (PLD2) has a key role in breast cancer formation and metastasis formation with PLD small inhibitors reducing primary tumor growth. This study aimed to evaluate the importance of targeting PLD on the tumor microenvironment. We provide evidence about the beneficial effect of PLD inhibitors [FIPI (dual PLD1/PLD2) or VU0155072-2 (PLD2 inhibitor)] on avoiding infiltration of tumor-helping macrophages and neutrophils. Tumor growth and metastasis within the primary tumors had low (<20% over controls) PLD enzyme activity. Unexpectedly, we found that the inhibitors also affected PLD2 gene expression and protein albeit at a lesser extent. The later could indicate that targeting both the actual PLD enzyme and its activity could be beneficial for potential cancer treatments *in vivo*. F4/80 and Ly6G staining of macrophages and neutrophils, respectively, and Arg1 staining data were consistent with M2 and N2 polarization. NOS2 staining increased in xenotransplants upon treatment with PLD2 inhibitors suggesting the novel observation that an increased recruitment of M1 macrophages occurred in primary tumors. PLD inhibitor-treated primary tumors had large, fragile, necrotic areas that were Arg1^+^ for M2 macrophages. The xenotransplants also caused the formation of large F4/80^+^ and Ly6G^+^ (>100 μm) clusters in lungs. However, PLD inhibitors, particularly FIPI, were able to diminish leukocyte presence. *Ex vivo* chemotaxis and PLD activity of peripheral blood neutrophils (PMN) and peritoneal macrophages was also determined. Whereas PMN had impaired functionality, macrophages did not. This significantly increased (“emboldened”) macrophage function was due to PLD inhibition. Since tumor-associated leukocytes in primary tumors and metastases were targeted via PLD inhibition, we posit that these inhibitors have a key role in cancer regression, while still affording an appropriate inflammatory response at least from off-site innate immunity macrophages.

## Introduction

Macrophages and neutrophils have been implicated in many studies of human breast cancer with a growing emphasis currently being placed on the study of inflammatory breast cancer (IBC), whereby leukocytes isolated from the tumor microenvironment of such patients secrete cytokines involved in cell movement, which contributes to propagation and metastatic spreading of IBC cells [1,2]. Both macrophages and neutrophils are associated with poor prognosis in breast cancer studies [3,4]. Neutrophils are recognized as both being inhibitors and promoters of cancer, as they can eliminate tumor cells disseminated from the main tumor but also prime the seeding of metastatic cells in the lung. Macrophages and neutrophils that are in the closest proximity to breast tumors are termed tumor-associated macrophages (TAM) and tumor-associated neutrophils (TAN). TAMs and TANs are further subdivided into M1 (“classical”) or M2 (“alternatively activated”) macrophages or N1 or N2 neutrophils, respectively, which represent either anti-tumoral (classified with the number 1) or pro-tumoral (classified with the number 2) properties dependent upon responses to growth factors, cytokines and chemokines, as well as proteases [2,5].

The transition from M1 or N1 phenotypes to that of M2 or N2 phenotypes indicates an overall subcellular change in the tumor microenvironment [2]. Such changes involve a significant switch in the orientation/polarization and differentiation of recruited mononuclear phagocytes that ultimately commandeer the local innate immune system away from its original anti-tumor functions to that of a pro-tumor environment [2]. Expression of proteolytic activities in TAMs and TANs from pre-invasive tumors forces the basement membrane to degrade and breakdown to the point that these types of aggressive tumor cells escape from the initial tumor and invade into the surrounding stroma and beyond into other tissues [6]. TAMs and TANs can be detected immunohistochemically using antibodies specific to many different macrophage- (F4/80 and arginase 1 (Arg1)) or neutrophil-specific (Ly6G) proteins [7–10].

TAMs and TANs secrete growth factors that promote tumor growth and metastasis [11]. Depletion of TANs or TAMs has been shown to slow down tumor growth [4,12–14]. TAMs secrete various growth factors, e.g. vascular epidermal growth factor (VEGF), platelet-derived growth factor (PDGF) and transforming growth factor β (TGF-β) [15–17]. TAMs also secrete epidermal growth factor (EGF) in response to macrophage colony-stimulating factor (MCSF), which is released by cancer cells and helps them proliferate [18]. Neutrophils are attracted to the tumor microenvironment by IL-8 that is secreted by human tumor cells [19–21]. Cells in the tumor microenvironment biologically resemble the functions of inflammation and wound healing [22,23]. As such, targeting the diverse aspects of the tumor microenvironment during cancer treatment in association with targeted immune suppression is a significant clinical goal.

Another protein that has a key role in macrophage and neutrophil signaling is phospholipase D (PLD) [24,25]. Several studies have implicated PLD in cancer cell transformation and progression [26–28]. The isoform phospholipase D2 (PLD2) enhances cell invasion both *in vitro* and *in vivo*, as does the other isoform phospholipase D1 (PLD1) [29–31]. PLD2 positively influences secretion of chemoattractant IL-8 [19–21,32,33]. Increased phospholipase D expression and increased PLD activity has been documented in human gastric, colorectal, breast, renal and papillary thyroid cancers [34–38]. Overexpression of lipase-active phospholipase D2 (PLD2) in lymphoma cells was found to be important for tumorigenesis of liver metastases to occur *in vivo*, while overexpression of enzymatically-inactive PLD2 hindered liver metastases [30]. The vascularization of melanoma or lung tumors from phospholipase D1 (PLD1) knockout mice was significantly reduced as a result of PLD downregulation in the tumor environment [29]. Additionally, knockdown of PLD2 in an aggressive cell line of human breast cancer using short-hairpin RNA (shRNA) specific for PLD2 decreased primary mammary tumor growth and lung metastases in a xenotransplanted SCID mouse model [31]. Stable transduction of either PLD1 or PLD2 overexpression in a much less aggressive human breast cancer cell line boosted tumor growth in a SCID model [31].

There are multiple modes of interaction between PLD and certain of its small molecule inhibitors, such that different binding sites on PLD2 are in play when PLD2 is bound to irreversible versus partially competitive inhibitors [39]. Such a repertoire of binding sites encompasses both PLD2 catalytic and regulatory regions and suggest mixed inhibition outcomes. PLD inhibitors delivered to a SCID model were effective in significantly delaying both the onset and size of primary breast tumors and the number of resulting metastatic axillary tumors [31].

We undertook the current study to ascertain a possible role of PLD in TAMs and TANs. We report herein that both TAMs and TANs were present in an orthotopic murine model xenotransplanted with the highly invasive MDA-MB-231 human breast cancer cells. Additionally, we have found further that the tumor promoting effects in leukocytes were abolished via inhibition of PLD through the use of small-molecule inhibitors specific for PLD, which we hypothesize indicates that the inflammatory PLD has a key role in breast cancer progression. This could potentially expand upon important information about the beneficial effects of this class of inhibitors in breast cancer.

## Materials and Methods

### Antibodies and reagents

Rabbit anti-PLD2 (H-133) and Mouse anti-Arg1 (E-2) IgG antibodies were from Santa Cruz Biotechnology (Santa Cruz, CA). Rat anti-mouse F4/80 AlexFluor488 IgG (cat. # MF48020), rat anti-mouse Ly6G R-PE IgG (cat. # RM3004), Rat anti-mouse IgG2a R-PE isotype control (cat. # R2B04) and rat anti-mouse IgG2b-FITC isotype control (cat. # R2B01) antibodies were from Invitrogen (Carlsbad, CA). The F4/80 and Ly6G antibodies positively stain F4/80^+^ macrophages or predominantly stain Ly6G^+^ neutrophils (and to a much lesser extent, ~7% of total signal could be staining for Ly6C+ monocytes), respectively. Mouse anti-PLD2 (SKB2) IgG antibody was from EMD Millipore (Billerica, MA). Tissue paraffin embedding, sectioning and mounting and H&E staining were performed by AML Laboratories, Inc. (Baltimore, MD). EZ-DeWax Solution was from BioGenex (Fremont, CA). Vector hematoxylin nuclear counterstain (Gill’s formulat), VectaMount AQ and VectaShield Mounting media were from Vector Laboratories (Burlingame, CA). Transwells were from Corning, Inc. (Tewkesbury, MA). Fetal calf serum was from Gemini Bio-Products (Sacramento, CA). MDA-MB-231 human breast cancer cells were from ATCC (Manassas, VA). 5-fluoro-2-indolyl des-chlorohalopemide (FIPI) and N-[2-(4-oxo-1-phenyl-1,3,8-triazaspiro[4,5]dec-8-yl)ethyl]-2-naphthalenecarboxamide (VU0155072-2) were from Cayman Chemical (Ann Arbor, MI) and were used according to [31,40–42] ([VU0155072-2 has also referred to as “NOPT” [31,40–42]]; Alzet micro-osmotic pumps were from Durect Corp. (Cupertino, CA).

### Animal experiments

All animal procedures and housing occurred in a facility accredited by AAALAC International, and all experimental procedures involving animals were reviewed and approved by Wright State University’s Institutional Laboratory Animal Care and Use committee. Eight week old female B- and T lymphocyte-deficient SCID/CB17 mice (C.B117/IcrHsd-Prkdc-Scid) were purchased from Harlan Laboratories (Indianapolis, IN). Mice were given a week to acclimate to the animal facility before they were studied. To minimize the risk of any exogenous infection, the SCID mice were maintained and cared for in sterile, static micro-isolation cages. Mice received irradiated food (Harlan Teklad 2920X, Harlan Laboratories) and sterile water *ad libitum*.

### Humane endpoints were in place during the animal study; method of euthanasia used in the study; and frequency of animal monitoring and well-being

Regarding the mice to be used for experimental purposes that received xenotransplantation of human breast cancer cells, the end-point criteria were: *(a)* body weight loss over 20% due to lack of proper nourishment; *(b)* tumor size in excess of 1 cm in 45 days; or *(c)* ulceration of the initial mammary tumor greater than 1 cm in diameter. In all 3 cases, any of the animals that met these criteria would be removed from the study and euthanized by certified personnel at WSU LAR or by Karen M. Henkels in the PI’s lab (Dr. Julian G. Cambronero). Euthanasia was performed by CO2 inhalation followed by cervical dislocation. Mice were checked daily by WSU LAR staff and an additional time each day by the PI’s lab when a pair of investigators would work together in tandem. Specific signs used to assess animal health, body condition and overall well-being included: was there evidence that food/water were being consumed or not and if the animals maintained/gained body weight or not, was the animal well-groomed with a smooth coat or showing signs of poorly groomed rough coat, was the animal shivering, was there discharge from the animal eyes, was the animal energetic or lethargic, did the animal move around in its cage or remain isolated in only one place in the cage, were there any signs of bleeding or ulceration from the site of the primary tumor or the Alzet pump (see below) site, did the animal appear to be in pain, etc. Additionally, the size of the primary tumors was measured on a daily basis using digital calipers, which required that each and every mouse used in the study was directly handled and its health thoroughly assessed by the pair of investigators from the PI’s lab (Dr. Cambronero). Any animals that failed these criteria were then further assessed by the veterinarian and treated if possible or euthanized if not possible.

### Implantation of Alzet miniature osmotic pumps

In experiments studying the effects of PLD inhibitors, Alzet miniature osmotic pumps (Durect Corp., Cupertino, CA) were used as an inhibitor-delivery system. The pumps were aseptically filled with either the vehicle (50% DMSO) or inhibitory compound at a concentration that would deliver 1.8 mg/kg/day of 5-fluoro-2-indolyl des-chlorohalopemide (FIPI) and N-[2-(4-oxo-1-phenyl-1,3,8-triazaspiro[4,5]dec-8-yl)ethyl]-2-naphthalenecarboxamide (VU0155072-2) at a rate of 0.11 μl/hr for 4–5 weeks. For FIPI, this concentration is equivalent to ~300 nM given a 5.5 h half-life and 18% bioavailability [40], which exerts full inhibition on PLD2 [41]. For VU0155072-2, the current half-life is not available from the literature, but it should be sufficient to inhibit PLD2, especially since the IC_50_ for PLD1 is in the micromolar range and is much greater than PLD2 [42]. Therefore, 1.8 mg/kg/day for each inhibitor should yield significant inhibition of PLD or tyrosine kinases. Filled pumps were equilibrated in sterile 0.9% saline at room temp for 18 h to reach steady state and optimal pump performance prior to implantation. After equilibration, pumps were aseptically implanted subcutaneously on the left dorsal thoracic area of the SCID mice while under isofluorane anesthesia. Carprofen (5 mg/kg) was administered subcutaneously once for analgesia following surgery.

SCID mice were orthotopically xenotransplanted one day after implantation with 4–6 x 10^6^ cells MDA-MB-231 in sterile HBSS + 0.5% BSA in the left mammary fat pad with the mice under isoflurane anesthesia. Approximately 4 weeks after injection of cancer cells, mice were humanely euthanized. The primary breast tumor, lung, any metastatic axillary tumors and livers were surgically excised and final dimensions measured. The tissues were then fixed and stained with hematoxylin and eosin.

### Real-time (Quantitative) Reverse Transcription-PCR

Total RNA was isolated from MDA-MB-231 cells with the RNeasy minikit. RNA concentrations were determined by fluorometric assay with the Ribogreen RNA Quantitation Kit, and samples were normalized to 20 ng/ml RNA. Reverse transcription was performed with 210 ng of RNA, 210 ng of random hexamers, 500 μM dNTPs, 84 units of RNase OUT and 210 units of Moloney murine leukemia virus reverse transcriptase and incubated at 42°C for 55 min. Quantitative PCR reactions were run with 100 ng of total input RNA (5 μl) and 1 μl of the PLD2 gene expression assay (6-carboxyfluorescein (FAM)-labeled) with the final concentrations being 200 and 400 pmol for the primers and probe, respectively. Sequences for primers and fluorescent probes, synthesized by Invitrogen, are: PLD1, FAM-5’-ACGAAAAGCACAACAAGGAGTGAGG-3’; PLD2, FAM-5’-TTGGCCCGGTGGTTTGTGAATGGGG-3’. Quantitative PCR conditions for the Stratagene Cycler were: 95°C for 3 min and then 50 cycles of the next 3 steps: 30 s 95°C, 1 min 60°C, and then 1 min 72°C. The “cycle threshold” *C*t values were chosen from the linear part of the PCR amplification curve where an increase in fluorescence can be detected at >10 S.E. above the background signal. Δ*C*t was calculated as: Δ*C*t = Avg. PLD *C*t—Avg. Housekeeping *C*t, and gene -fold expression was calculated as 2^-(ΔΔ*C*t)^ = 2^-(experimental condition Δ*C*t—control Δ*C*t)^.

### PLD activity assay

Samples were processed for PLD2 activity in PC8 liposomes and [^3^H]n-butanol beginning with the addition of the following reagents (final concentrations): 3.5 mM PC8 phospholipid, 45 mM HEPES (pH 7.8), and 1.0 μCi [^3^H]n-butanol in a liposome form, as indicated in [43]. Samples were incubated for 20 min at 30°C with continuous shaking. Addition of 0.3 ml ice-cold chloroform/methanol (1:2) stopped the reactions. Lipids were then isolated and resolved by thin layer chromatography. The amount of [^3^H]-PBut that co-migrated with PBut standards was measured by scintillation spectrometry.

### Immunohistochemical analyses of mouse primary breast tumor, lung and liver tissues using immunofluorescence staining

Each slide was incubated 2x in BioGenex brand EZ-Dewax solution for 5 min each incubation and then washed 3x with distilled water. Sections were blocked with 10% FCS, 0.1% Triton X-100 in TBS-T for 1 hr. at room temp. Typically, sections were incubated in primary antibody using 2ml per slide at a 1:200 dilution in blocking buffer for 1 hr. at room temp and were then washed 3x with PBS for 5 min each wash. Next, sections were incubated in a secondary fluorescently-labeled antibody using 2ml per slide at a 1:200 dilution in blocking buffer for 1 hr. at room temp and were then also washed 3x with PBS for 5 min each wash. For reference, sections were stained with 1:2000 DAPI for 5 min. at room temp to visualize nuclei in regards to other cellular components of interest and were rinsed 1x with PBS and then were mounted using VectaShield Mounting Media. Immunofluorescence microscopy was performed using a Leica DMI 6000B inverted fluorescent microscope.

### Immunohistochemical analyses of mouse primary breast tumor, lung and liver tissues using DAB chromogen staining

Each slide was incubated 2x in BioGenex brand EZ-Dewax solution for 5 min each incubation and then washed 3x with distilled water. The HRP-DAB cell and tissue staining kit was used from R&D Systems (Minneapolis, MN) to block the sections, incubate with HSS-HRP and to stain with DAB chromogen per the manufacturer’s quidelines. Typically, following sequential blocking steps, sections were incubated in biotinylated rat anti-mouse primary antibody (either F4/80 or Ly6G) using 2ml per slide at a 1:200 dilution in blocking buffer (10% FCS, 0.1% Triton X-100 in TBS-T) for 1 hr at room temp and were then washed 3x with PBS for 15 min each wash. Next, sections were incubated in HSS-HRP from the HRP-DAB cell and tissue staining kit using 1–3 drops per slide for 30 min at room temp and were then also washed 3x with PBS for 2 min each wash. Sections were stained with 1–5 drops of DAB chromogen solution from the kit (2 drops DAB per 2 ml of DAB chromogen buffer) for 1 min. Sections were rinsed with distilled water for 5 min and then counterstain with Vector hematoxylin nuclear counterstain—Gill formula (Vector Laboratories, Burlingame, CA) for 1 min. Sections were rinsed with distilled water 3x for 5 min each wash, dipped 10x in acid rinse solution (2 ml glacial acetic acid + 98 ml distilled water), dipped 10x in tap water, incubated for 1 min in bluing solution (1.5 ml 30% NH_4_OH + 98.5 ml 70% ethanol) followed by 10 dips in tap water. Sections were mounted using VectaMount AQ Mounting Media. Brightfield microscopy was performed using a Nikon Diaphot inverted microscope.

### Quantification of immunofluorescence staining in various tissues

We used ImageJ software to quantify the levels of immunofluorescence staining for the selected target proteins based on if the tissue was from primary or axillary metastatic tumors versus lung or liver tissues. These results were quantified using two different criteria. Protein levels in primary or metastatic axillary tumors were quantified as “total mean target protein fluorescence” (as total readout of fluorescence intensity in the field of view) in terms of arbitrary units. Alternatively, metastatic burden in the xenotransplanted mouse lungs and livers were determined by quantifying the area (μm^2^ x 10^3^) of metastatic “foci” present per field of view in the various lung and liver sections stained with PLD2, F4/80, Ly6G or Arg1 antibodies. Using either quantification strategy, triplicate fields were counted per each section and are represented as mean ± SEM. When appropriate, different views of different biological replicates are shown. In those quantifications the background isotype controls have been subtracted.

### Chemotaxis of mouse peritoneal macrophages (MΦ) and peripheral blood neutrophils (PMN)

Seven to eight ml of peripheral blood from ~10 mice was used to purify PMNs using a Ficoll gradient. MΦ were obtained from peritoneal lavage of xenotransplanted mice. Cells were resuspended at a density of 5 x 10^5^ cells/ml in chemotaxis buffer (DMEM with 0.1% BSA). A total of 200 μl was placed in the upper chambers (or inserts) of transwell inserts (5 μm pore for chemotaxis of PMNs or 8 μm pore for MΦ) that were separated from the lower wells by a 8 mm diameter polycarbonate membrane. Chemoattractants were prepared fresh the day of the experiment in 1x PBS–0.1% BSA, pH, 7.2, at a stock concentration of 1 μM. When ready for the assays, chemoattractants were diluted to working concentrations of either 30 nM MCP-1 for MΦ or 30 nM fMLP for PMNs in 500 μl of chemotaxis buffer and placed into the lower wells of 24-well plates. Chemotaxis inserts were incubated for 1–3 hr at 37°C under a 5% CO_2_ atmosphere. The number of cells that migrated to the lower wells was calculated by counting four fields in duplicate.

### Statistical Analyses

Data presented in the Figs as bars are means + Standard Error of the Mean (SEM) (standard deviation/n1/2, were n is the sample size). Experiments were performed in technical triplicates (for qPCR assays) or technical duplicates (for PLD activity assays) for n = 5 independent experiments. The difference between means was assessed by the Single Factor Analysis of Variance (ANOVA) test, calculated using SigmaPlot version 10 (Systat Software Inc, San Jose, CA). Probability of p<0.05 indicates a significant difference. In the Figs, the (*) symbols above bars denote statistically significant (P<0.05) ANOVA increases between samples and controls. The (#) symbols above bars denote statistically significant (P<0.05) ANOVA decreases between samples and controls. ImageJ quantification of the negative effect of PLD inhibitors on different tissues for immunofluorescence microscopy considered two values: total mean fluorescence of field of view and the total area of positive foci for n = 5 each condition. Data for these quantifications are presented in the Figs as bars are means + Standard Error of the Mean (SEM).

## Results

### Effect of small-molecule PLD inhibitors on PLD2 in breast tumors following xenotransplantation of human breast cancer cells

**Fig 1:**
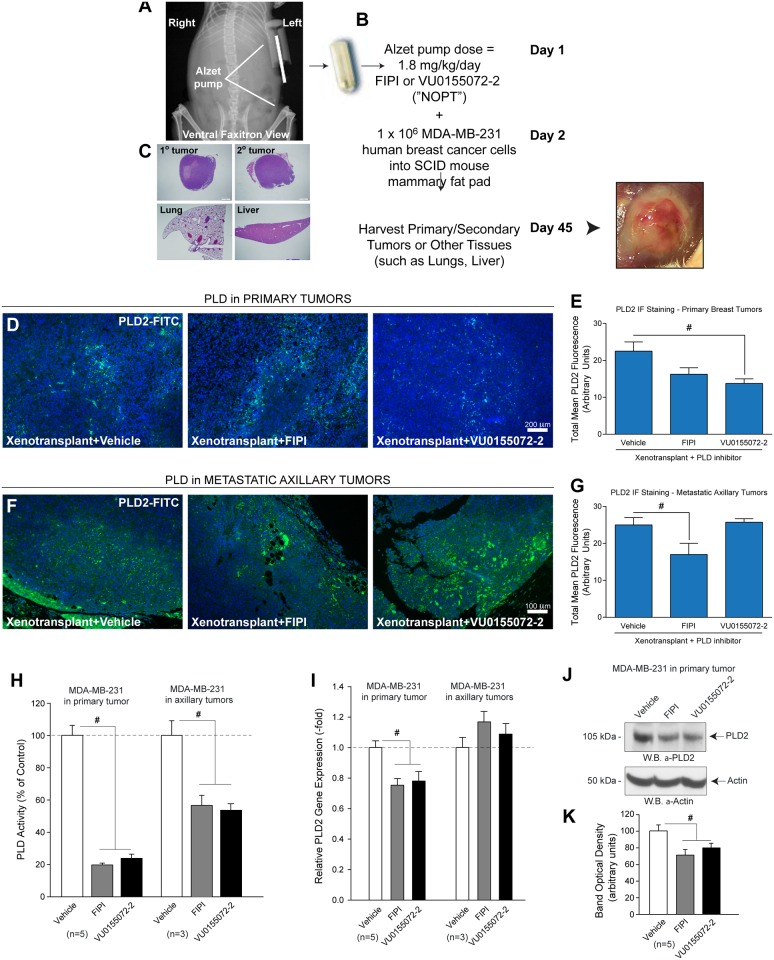
Inhibitory effects of FIPI or VU0155072-2 “NOPT” in mouse mammary tumors following xenotransplantation of human breast cancer cells. (**A-C**) SCID mice were xenotransplanted with human MDA-MB-231 breast cancer cells following implantation of Alzet pumps containing 2 different PLD-small molecule inhibitors of activity: 5-fluoro-2-indolyl des-chlorohalopemide (FIPI) or N- [2- (4- oxo- 1- phenyl- 1, 3, 8- triazaspiro[4, 5]dec- 8- yl)ethyl]- 2- naphthalenecarboxamide (VU0155072-2) [also referred to as “NOPT” [31,40–42]]. Approximately 45 days post xenotransplantation, primary and axillary breast tumors, lungs and livers were harvested from mice that received MDA-MB-231 cancer cells and fixed in formalin, which were then embedded in paraffin, sectioned and mounted onto glass microscope slides. (**D,E**) Negative effect of small molecule inhibitors on PLD2 in mouse primary mammary and metastatic axillary tumors. (**F,G**) Images were quantified in terms of total mean fluorescence per field of view for PLD2 using ImageJ software. (**H**) PLD enzyme activity and (**I**) PLD2 gene expression. (**J**) PLD2 protein expression and (**K**) densitometry of same Western blot. Results are mean relative PLD gene expression (-fold) ± SEM, or mean PLD activity ± SEM, in terms of percent of control (100% = 1,254 ± 117 dpm/mg protein). Sample sizes were n = 5 for each set of animals used and 6–8 fields were viewed for each section. Statistical analyses for the increases (*, p<0.05, ANOVA) in xenotransplants and the decreases (#, p<0.05, ANOVA) in PLD inhibitors-treated samples.

In a previous study (31), we have provided *in vivo* evidence that the PLD2 isoform is fundamental to human breast cancer progression. We wanted to test the ability of PLD activity inhibitors to affect breast cancer tumor growth and their metastasis, as well as the effect, if any, on the tumor microenvironment. We chose to deliver PLD inhibitors continually using micro-osmotic pumps implanted subcutaneously in SCID mice. As summarized in the scheme presented in Fig 1A and 1B, micro-osmotic pumps were aseptically filled with either the vehicle (50% DMSO) or inhibitory compound at a concentration that would deliver 1.8 mg/kg/day of the PLD-specific inhibitors 5-fluoro-2-indolyl des-chlorohalopemide (FIPI) and N-[2-(4-oxo-1-phenyl-1,3,8-triazaspiro[4,5]dec-8-yl)ethyl]-2-naphthalenecarboxamide (VU0155072-2) ([VU0155072-2 has also been referred to as “NOPT” [31,40–42]]; at a rate of 0.11 μl/hr for 45 days (Fig 1A and 1B). There are no cytotoxic effects of 750 nM or 7.5 μM FIPI or 1 μM VU0155072-2 on DNA or protein synthesis of keratinocytes *in vitro* [44].

Primary and secondary tumors and organs (lungs and livers) were collected and processed for relevant analyses (Fig 1C). Primary breast tumor sections revealed slight decreases in PLD2 staining in the primary breast tumor tissue sections from the samples that received FIPI or VU0155072-2 when compared to vehicle-treated samples (Fig 1D), which is quantified in Fig 1E. In secondary axillary tumors (Fig 1F), only FIPI-treatment showed a significant decrease in PLD staining, which is quantified graphically in Fig 1G. We posit that the detected inhibition on tumor growth and metastasis could be likely due to reduced lipase activity within the tumors/metastases (Fig 1H–1K). The level of enzyme activity fell to ~20% over controls in primary tumors and to ~55% in axillary tumors (Fig 1H). Unexpectedly, we found that the inhibitors also affected both gene (Fig 1I) and protein expression (Fig 1J and 1K), albeit at a lesser extent than that of the enzyme activity (~70% levels over controls). This finding was confined to primary tumors, i.e., no statistical effect in secondary (axillary) tumors for gene expression (Fig 1I). Therefore, the inhibitors affected PLD activity *in vivo* and also had a collateral (smaller) effect on reducing expression levels. The later was not expected and it would indicate that targeting both the actual enzyme and the activity could be beneficial for potential cancer treatments *in vivo*.

### Effect on tumor volume and lung metastasis

**Fig 2:**
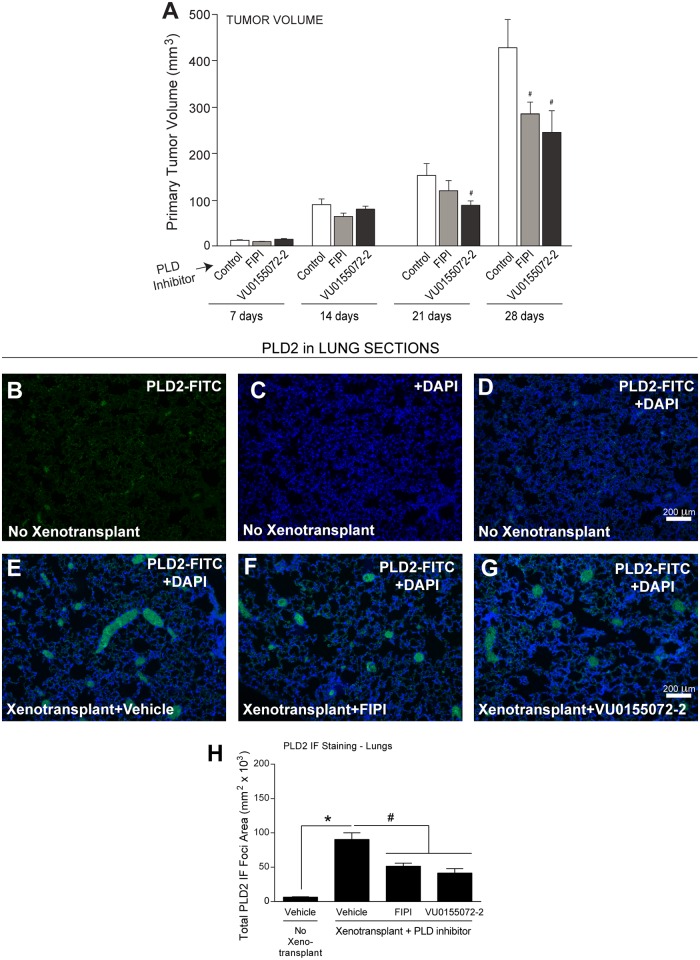
PLD inhibitors decrease PLD2 expression in foci of lungs following xenotransplantation. (**A**) Primary tumor volume from xenotransplanted mice in the presence or absence (“control” = vehicle only) of PLD inhibitors delivered by Alzet pumps, as in Fig 1 legend. (**B-D**) Lung sections from non-xenographed controls. (**B**) PLD2-FITC staining only. (**C**) DAPI staining only and (**D**) the merged PLD2-FITC+DAPI staining. (**E-G**) Lung tissues from xenotransplanted mice. (**E**) Xenotransplanted controls with Alzet pumps delivering vehicle. (**F**) Xenotransplanted with Alzet pumps delivering vehicle FIPI or (**G**) VU0155072-2. Scale bar = 200 μm. (**H**) ImageJ quantification of the negative effect of PLD inhibitors on total mean fluorescence of the total area of PLD2 positive foci in lung tissues in terms of μm^2^ x 10^3^. Sample sizes were n = 5 for each set of animals used and 6–8 fields were viewed for each section. Statistical analyses for the increases (*, p<0.05, ANOVA) in xenotransplants and the decreases (#, p<0.05, ANOVA) in PLD inhibitors-treated samples.

The lack of PLD activity in the inhibitor-treated samples shown in Fig 1I also affected the volume of the primary tumors, especially 21 days post-transplant and later (Fig 2A). We surmised that: *(i)* if PLD2 was crucial to migration of xenotransplanted breast cancer cells from the site of xenotransplantation into the circulatory system and beyond into other tissues that have been metastasized (i.e. lungs) and, *(ii)* if PLD-specific inhibitors reversed this migration, then we would expect to observe a relative decrease in the levels of PLD2 within such tissue. We confirmed the presence of PLD2-positive foci in lungs from mice that were xenotransplanted (Fig 2E–2G) compared to controls (Fig 2B–2D), which is quantified in Fig 2H. PLD2 was detected throughout the mouse lung as very small punctate foci. We consider this to be the basal level of PLD2 expression in the mouse lungs and the increased levels of PLD2 expression observed in the xenotransplanted samples (Fig 2E and 2G) were detected as larger areas of more profuse staining, which could be indicative of PLD2-containing tumor cells metastasizing to the lungs. Additionally, we observed a significant decrease in the area of PLD2-positive foci in lungs from xenotransplanted mice that received the PLD inhibitors compared to the controls.

### Tumor-associated macrophage (TAM) F4/80 and tumor-associated neutrophil (TAN) Ly6G proteins are present in mouse tumors/tissues following xenotransplantation and are reduced with PLD inhibitors

**Fig 3:**
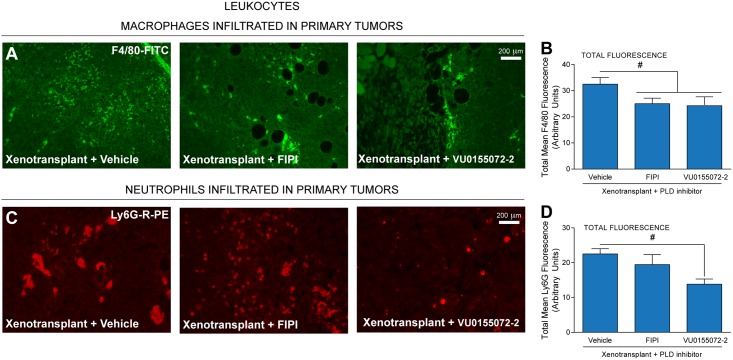
Presence of tumor-associated macrophage (TAM) F4/80 and tumor-associated neutrophil (TAN) Ly6G in xenotransplanted mice are suppressed by PLD inhibitors. (**A,C**) Immunofluorescence staining of mouse primary breast tumor sections using either a rat anti-mouse F4/80 AlexaFluor 488 IgG antibody specific for TAMs (**A**) or a rat anti-mouse Ly6G-R-PE IgG antibody specific for TANs (**C**). Each set of three panels represents results from Xenotransplanted MDA-MB-231 cells in the presence of vehicle, FIPI or VU0155072-2, respectively. Scale bar = 200 μm. (**B,D**) ImageJ quantification of the negative effect of PLD inhibitors on the total mean fluorescence of each field of view for either F4/80 (**B**) or Ly6G (**D**) in primary metastatic breast tumors. Sample sizes were n = 5 for each set of animals used and 6–8 fields were viewed for each section. Statistical analyses for the increases (*, p<0.05, ANOVA) in xenotransplants and the decreases (#, p<0.05, ANOVA) in PLD inhibitors-treated samples.

We next investigated the effect, if any, of PLD inhibitors on the tumor microenvironment with a specific focus on certain components of the circulatory/inflammatory system that are affected by PLD signaling, such as macrophages or neutrophils, both of which have been implicated in poor prognosis of breast cancer studies [3,4]. We found tumor-associated macrophages (TAM) and tumor-associated neutrophils (TAN) that were positive for F4/80 (a macrophage-restricted cell surface glycoprotein) (Fig 3A and 3B) and LY6G antibodies (a myeloid differentiation antigen that is predominantly expressed in neutrophils and to a much lesser extent in monocytes) (Fig 3C and 3D). Importantly, there was a significant decrease in the total mean fluorescence of F4/80 and Ly6G staining in primary mammary tumors. This decrease was larger in Ly6G-stained tissues (possible from TAN-specific staining) (~40%) (Fig 3D) than in F4/80-stained tissues (possible from TAM-specific staining, which is confirmed in subsequent figures) (~25%) (Fig 3B) and in either case, the decrease in accumulation of leukocytes was larger than the decrease in immunofluorescence area afforded by the inhibitors (Fig 2A). These data indicate that PLD inhibitors have a double effect: one that acts directly on tumor growth and another that is more pronounced that inhibits the accumulation of TAMs and TANs in the microenvironment.

**Fig 4:**
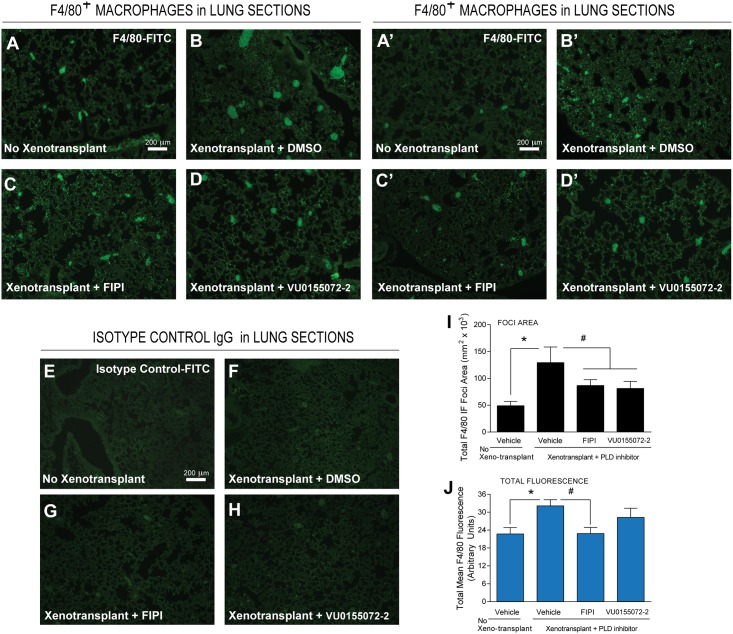
Decreased F4/80^+^ macrophages in the presence of PLD inhibitors using IF microscopy of lung sections. Lung sections from MDA-MB-231 xenotransplanted mice were incubated with a FITC-F4/80 IgG antibody specific for TAMs (**A-D**) or biological duplicates from independent experiments (**A’-D’**) or with an isotype control-FITC IgG antibody (**E-H**). (**A,A’,E**) Tissues from non-xenographed controls; (**B,B’,F**) from Xenotransplanted controls with Alzet pumps delivering vehicle; (**C,C’,G**) Xenotransplanted with Alzet pumps delivering vehicle FIPI or (**D,D’,H**) VU0155072-2. Scale bar = 200 μm. ImageJ quantification on the total area of positive foci for TAM using F4/80 staining (**I**) in lung tissues in terms of μm^2^ x 10^3^. Also, total mean fluorescence of each field of view is presented in panel (**J**). In both those quantifications the background isotype controls have been subtracted. Sample sizes were n = 5 for each set of animals used and 6–8 fields were viewed for each section. Statistical analyses for the increases (*, p<0.05, ANOVA) in xenotransplants and the decreases (#, p<0.05, ANOVA) in PLD inhibitors-treated samples.

**Fig 5:**
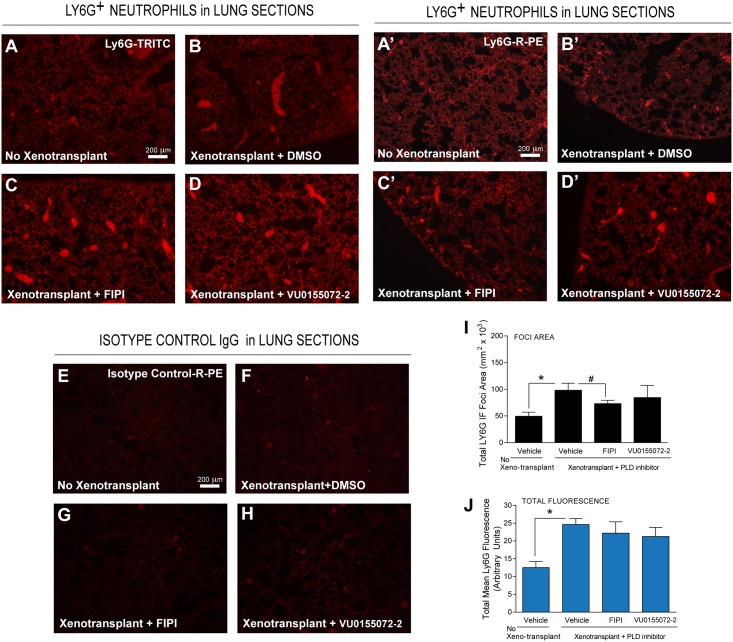
Decreased Ly6G^+^ neutrophils in the presence of PLD inhibitors using IF microscopy of lung sections. Lung sections from MDA-MB-231 xenotransplanted mice were incubated with a TRITC-Ly6G antibody specific for TANs (**A-D**) or biological duplicates from independent experiments stained with Ly6G-R-PE (**A’-D’**) TANs (**A-D**) or biological duplicates from independent experiments stained with Ly6G-R-PE or with an isotype control-R-PE IgG antibody (**E-H**). (**A,A’,E**) Tissues from non-xenographed controls; (**B,B’,F**) from Xenotransplanted controls with Alzet pumps delivering vehicle; (**C,C’,G**) Xenotransplanted with Alzet pumps delivering vehicle FIPI or (**D,D’,H**) VU0155072-2. Scale bar = 200 μm. ImageJ quantification on the total area of positive foci for TANs using Ly6G staining (**I**) in lung tissues in terms of μm^2^ x 10^3^. Also, total mean fluorescence of each field of view is presented in panel (**J**). In both those quantifications the background isotype controls have been subtracted. The use of TRITC- or R-PE labeling yielded virtually similar results. Sample sizes were n = 5 for each set of animals used and 6–8 fields were viewed for each section. Statistical analyses for the increases (*, p<0.05, ANOVA) in xenotransplants and the decreases (#, p<0.05, ANOVA) in PLD inhibitors-treated samples.

We next used IF staining specific for either mouse tumor-associated macrophages using a α-F4/80 antibody (Fig 4) or for mouse tumor-associated neutrophils using a α-Ly6G antibody (Fig 5). Xenotransplants caused the appearance of small (10–30) μm “punctae” and large (>100 μm) clusters (Figs 4B, 4B’, 5B and 5B’; compared to non-xenotranplats (Figs 4A, 4A’, 5A and 5A’). At present, we do not know the nature of these clusters, but suspect they could be due to metastases as well as non-specific cross-reactivity of nerve bundles in addition to large macrophage (Fig 4) or neutrophil (Fig 5) accumulations. Interestingly, lung slices probed with fluorophore-relevant isotype controls (Figs 4E or 5E) did not show the presence of those clusters, indicating that indeed, staining from the clusters in 4B,B’ is derived in large part from F4/80^+^ macrophages and in 5B,B’ are derived in large part from Ly6G^+^ neutrophils.

Moving now to the effect of PLD inhibitors, we found that in lung sections, there were fewer and smaller F4/80-stained “punctae” and “clusters” in FIPI- and VU0155072-2-treated samples (Fig 4C, 4D, 4C’ and 4D’) compared to vehicle (DMSO) alone, and a similar pattern for neutrophils was also found (Fig 5C, 5D, 5C’ and 5D’). This was quantified as foci area (Fig 4I) for F4/80^+^ macrophages and (Fig 5I) for Ly6G^+^ neutrophils; as well as total fluorescence in each field of view (Fig 4J) for F4/80^+^ macrophages and (Fig 5J) for Ly6G^+^ neutrophils. In either case there is decrease in cell staining of ~30–50% in xenotransplanted mice that received PLD inhibitors, particularly FIPI, indicating that the PLD inhibitors were able to diminish leukocytes associated to lung metastasis.

Taking all of this into consideration from the data presented in Figs 3–5, these results reinforce the importance of small-molecule inhibitors in reducing the presence of tumor-associated macrophages and neutrophils in tissues where breast cancer could invade and metastasize *in vivo*.

### The M2 macrophage protein Arg1 is present in mouse mammary tumors following xenotransplantation of human breast cancer cells

**Fig 6:**
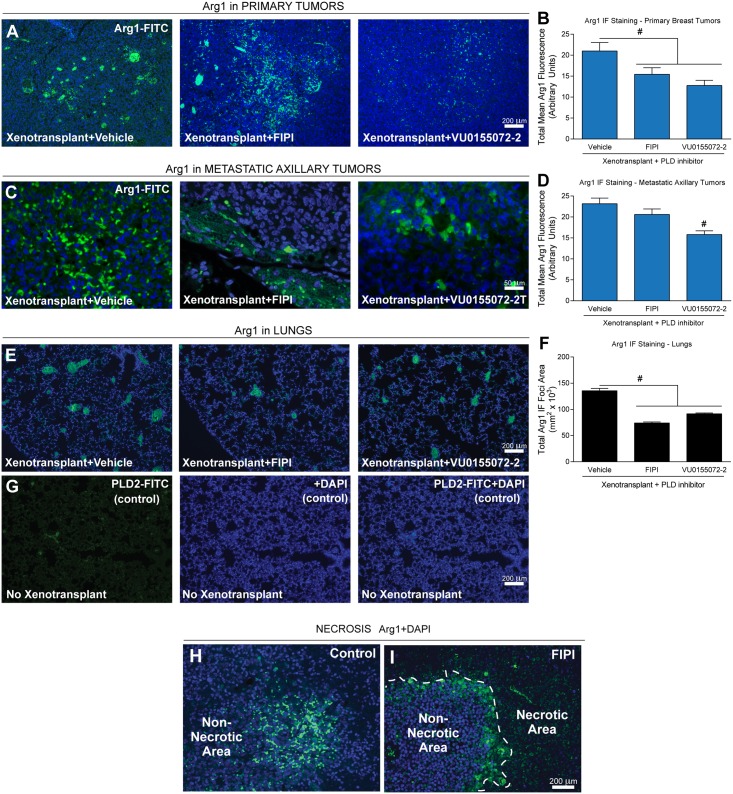
Induction of the M2 macrophage protein Arg1 in xenotransplanted mouse mammary tumors. (**A-B**) Mouse primary breast tumors. (**C-D**) metastatic axillary tumors. (**E-G**) Lungs showing xenotransplant vs. no-xenotransplant controls. Sections showing Arg1 expression. Nuclei were stained with DAPI for reference. Scale bars = 200 μm. (**B,D,F**) Quantification of the negative effect of PLD inhibitors on Arg1 staining. Sample sizes were n = 5 for each set of animals used and 6–8 fields were viewed for each section. Statistical analyses for the increases (*, p<0.05, ANOVA) in xenotransplants and the decreases (#, p<0.05, ANOVA) in PLD inhibitors-treated samples. **(H-I)** Presence of Arg1 in necrotic areas in xenotransplanted primary tumors. A boundary of non-necrotic versus necrotic cells within the primary tumors is delineated by the white, dashed line in the FIPI-treated sample **(I).**

TAMs promote tumorigenesis within the tumor microenvironment [45–47]. Arginine 1 (Arg1) is associated with M2 macrophage and N2 neutrophil polarization [2,10,48,49]. We determined if Arg1 was present in the primary or metastatic tumors in the xenotransplantation model. As shown in Fig 6, Arg1 was present in primary mammary tumors (Fig 6A and 6B) and metastatic axillary tumors (Fig 6C and 6D). Immunofluorescent staining from mice that had received PLD inhibitors was reduced when compared to xenotransplant+vehicle only samples. Although the data presented in Figs 4 and 5 using F4/80 and Ly6G staining are not necessarily specific for M2 macrophages or N2 neutrophils, the results shown in Fig 6 using staining of Arg1 are consistent for M2 macrophages or N2 neutrophils. Additionally, non-xenotransplanted mice generated absolutely no breast cancer-derived tumors under our experimental conditions. Consequently, there are no tissue sections of primary or secondary tumors available to include in this figure.

However, such analysis was performed for the lung sections, as non-xenotransplanted mice still had 2 lungs that could be metastasized. As such, we found that Arg1 (Fig 6E–6G) was similarly detected in lung tissue of xenotransplanted mice and then significantly decreased in both total Arg1 IF area and mean Arg1 fluorescence as a result of treatment with the PLD inhibitors. We also found that PLD-specific inhibitor-treated tumors had large, fragile, necrotic areas that were Arg1^+^ with M2 macrophages (Fig 6H and 6I). The presence of necrotic areas in PLD-specific inhibitor-treated samples (Fig 6I) was clearly more readily evident than in controls (Fig 6H). This could likely be due to the infiltration of Arg1^+^ M2-type macrophages to the sight of inflammation surrounding/within the tumor with the purpose of cleaning the inflamed site or to initiate resolution of the inflammation.

### Expression of NOS2 in primary breast tumors

**Fig 7:**
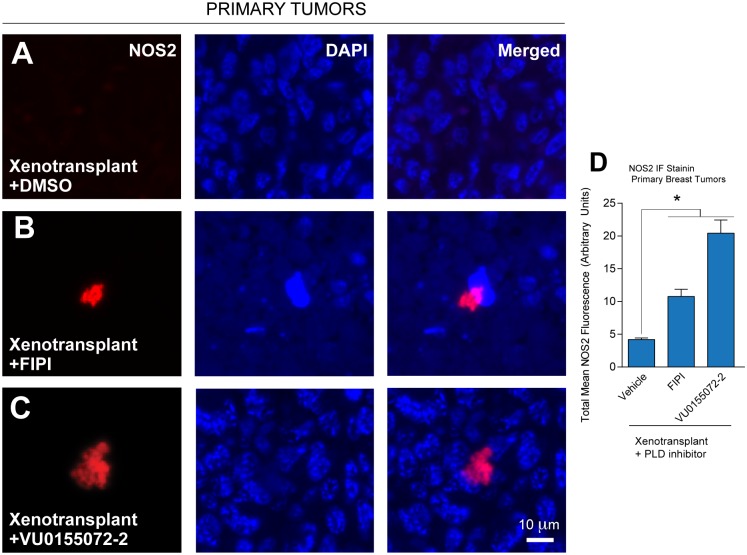
Lack of NOS2 induction in xenotransplanted mouse mammary tumors. (**A-D**) Primary breast tumor sections were incubated with rabbit anti-NOS2 IgG antibody followed by goat anti-rabbit IgG TRITC antibody and DAPI for reference. (**A**) Xenotransplanted MDA-MB-231 cells, vehicle. (**B** Xenotransplanted MDA-MB-231 cells, FIPI. (**C**) Xenotransplanted MDA-MB-231 cells, VU0155072-2. Scale bar = 100 μm. Sample sizes were n = 5 for each set of animals used and 6–8 fields were viewed for each section. (**D**) Quantification of NOS2 from IF images shown in (**A-C**). Statistical analyses for the increases (*, p<0.05, ANOVA) in xenotransplants and the decreases (#, p<0.05, ANOVA) in PLD inhibitors-treated samples.

Arginine 1 (Arg1) (associated with M2 macrophages and N2 neutrophils) metabolizes L-arginine to L-ornithine and urea and is in direct competition with nitrogen synthase 2 (NOS2). NOS2 is associated with M1 macrophages and is upregulated in the tumor microenvironment of TAMs and TANs and during tumor progression [50,51]. We found very little staining for NOS2 (Fig 7). Approximately, 1–2 positive findings every 6–8 fields studied was detected in primary tumor sections (red-stained spots). However, whether animals received inhibitor treatment or not resulted in obvious differences (Fig 7A vs. 7B and 7D), even though such examples of the rarity of these findings are presented. Data from Figs 6 and 7 suggest that Arg1 but not NOS2 was induced in primary and metastatic tumors and that Arg1 was then negatively affected by the presence of the PLD small-molecule inhibitors, which suggests that the presence of a TAM response could be involved in a non-classical immune response in this human breast cancer formation/metastasis model. In addition, the increased NOS staining in xenotransplanted mice upon treatment with PLD inhibitor (Fig 7) may suggest that these inhibitors increased recruitment of M1 macrophages to the tumor microenvironment.

These data suggest a shift in macrophage polarization from M2 to that of the less invasive and anti-tumoral M1 macrophage in the PLD-specific inhibitor-treated samples. Additionally, the use of PLD inhibitors could be a means to recruit M1 macrophages to the tumor microenvironment, which could yield a more favorable outcome in terms of patient health.

### The hepatic cellular environment changes concomitantly with tumorigenesis that is decreased by PLD inhibitors

**Fig 8:**
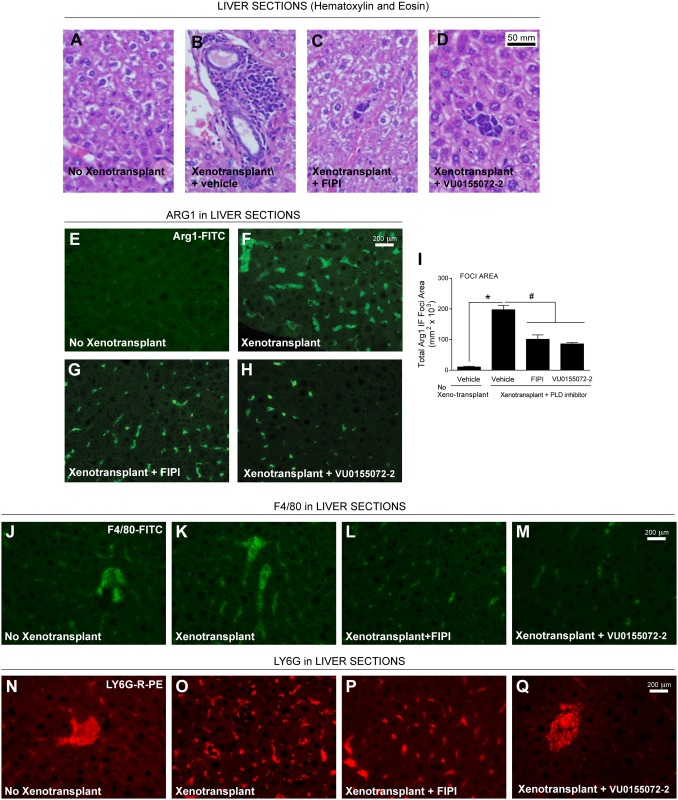
The hepatic cellular environment is altered with PLD inhibitors. Liver sections were incubated with hematoxylin and eosin (**A-D**) or antibodies specific for Arg1 (**E-H**), F4/80 (**J-M**) or LY6G (**N-Q**) for immunofluorescence microscopy. Xenotransplanted MDA-MB-231 cells in the presence of vehicle, FIPI or VU0155072-2, respectively, and no xenotransplantation in the presence of vehicle. (**A-D**) Scale bar = 50 μm. (**E-Q**) Scale bar = 200 μm. (**I**) ImageJ quantification of the negative effect of PLD inhibitors on the total area of Arg1 positive foci in liver tissues in terms of μm^2^ x 10^3^. Sample sizes were n = 5 for each set of animals used and 6–8 fields were viewed for each section. Statistical analyses for the increases (*, p<0.05, ANOVA) in xenotransplants and the decreases (#, p<0.05, ANOVA) in PLD inhibitors-treated samples.

As Arg1 is significantly expressed in the liver in general and as our previous results indicate the significance of Arg1 in tumor metastasis to lung tissue of the model, we next determined if there was an effect of PLD inhibitors on liver Arg1 in samples from our model. Using hematoxylin and eosin staining of liver tissue sections from our mouse models, we noted no significant lesions in livers from mice that were not xenotransplanted (Fig 8A), but we did observe the presence of very mild multifocal perivascular lymphocyte and neutrophil aggregates in mice that were xenotransplanted only (Fig 8B).

Additionally, in the livers from xenotransplanted mice in the presence of PLD inhibitors, we observed improved liver appearance based upon the presence of small clusters of round cells with moderate cytoplasm randomly scattered throughout the parenchyma that included a few areas of granulopoiesis in the parenchyma (Fig 8C and 8D). The H&E data suggest a changing environment within the hepatic parenchyma of mice that were xenotransplanted with human breast cancer perhaps as a result of an underlying immune response that coincides with tumorigenesis. This change in hepatic cellular environment was also noted with a large increase in the amount of Arg1 localized to the liver of xenotransplant only samples compared to non-xenotransplanted samples (Fig 8E–8H), which was significantly reduced in the presence of FIPI or VU0155072-2 (Fig 8I). Similar results were also observed for F4/80 and LY6G staining in liver tissues (Fig 8J–8Q).

### Effect of PLD inhibitors on the gene expression of selected cytokines

**Fig 9:**
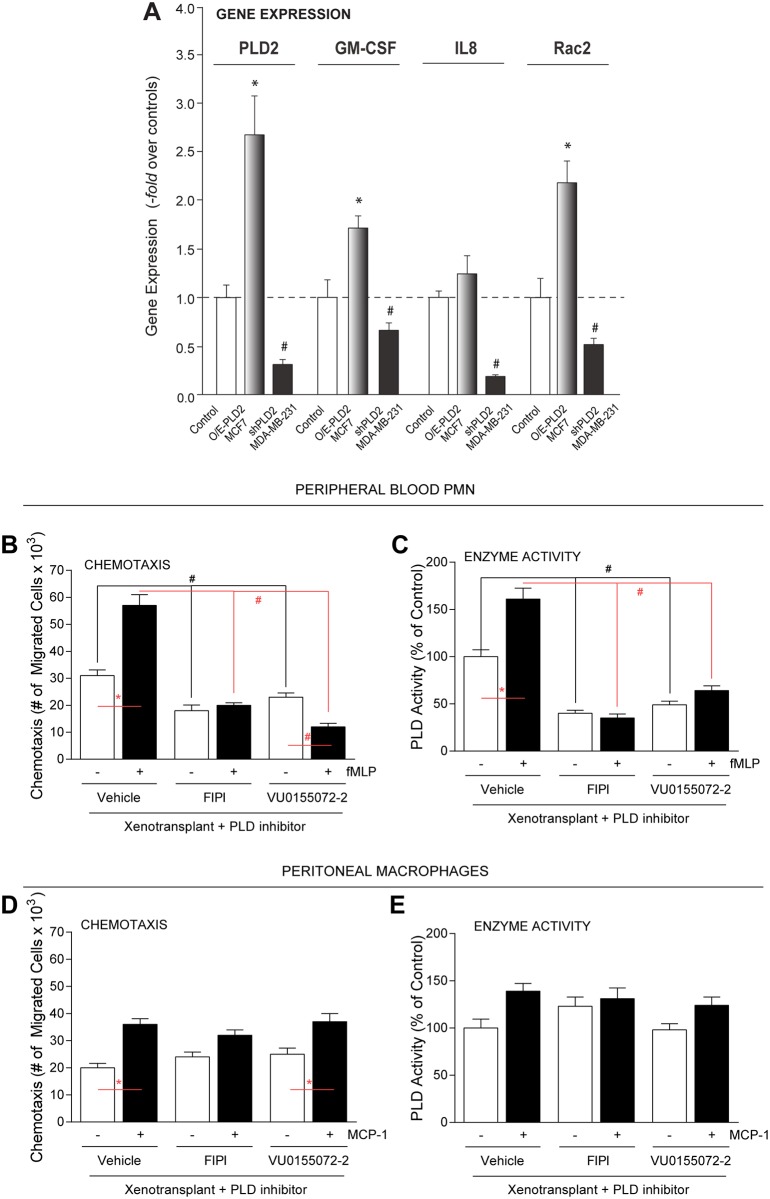
Differential effects of PLD small-molecule inhibitors on mouse leukocyte chemotaxis. (**A**) Gene expression by quantitative PCR of MCF-7 cells stably overexpressing (O/E) PLD2 (O/E-PLD2-MCF7) or MDA-MB-231 stably silenced with shRNA for PLD2 (shPLD2-MB-MB-231). Non-cancerous control cells, HMEC (human mammary epithelial cells). (**B-E**) Functional assay of non-tumor associated leukocytes. Peripheral blood PMNs (**B,C**) or peritoneal macrophages (**D,E**) from mice treated with either vehicle, FIPI or VU0155072-2 were used for chemotaxis assays (**B,D**) and PLD activity assays (**C,E**) in response to either 30 nM IL-8 (for PMNs) or 30 nM MCP-1 (for macrophages). For chemotaxis, the numbers of cells that migrated to the lower were calculated by counting four fields in duplicate. Triplicate results are mean ± SEM and are expressed in terms of either number of cell migrated per field or number of invading cells per insert. For PLD assay, the amount of TLC-isolated [^3^H]-PBut that co-migrated with PBut standards was measured by scintillation spectrometry. Results are mean ± SEM and are expressed in terms of percent of control. Statistical analyses for the increases (*, p<0.05, ANOVA) in xenotransplants and the decreases (#, p<0.05, ANOVA) in PLD inhibitors-treated samples.

Tumor-related tissues in xenotransplanted mice also experienced early stages of an inflammatory/immune response that coincided with severity of tumorigenesis. A possible mechanism would be an effect of the PLD inhibitors (through reducing the amount of PA available) on gene expression, as PA is a mitogen. We have shown previously [31] that these cells changed invasive phenotype, as shPLD2-MDA-MB-231 cells are less invasive than the parental MDA-MB-231 cells and O/E-PLD2-MCF-7 cells are much more invasive than the parental MCF-7 cells, both of which again highlight the key role of PLD in cancer cell invasiveness. We hypothesize that the level of specific cytokines could be altered in breast cancer cells following treatment with those inhibitors. Fig 9A indicates results of experiments using several human breast cancer cells lines prior to xenotransplantation. As shown in Fig 9A, PLD2, GM-CSF, IL-8 and Rac2 (which is important for cell chemotaxis and metastasis [52]) were significantly increased in PLD2-overexpressing MCF-7 cells and significantly decreased in PLD2-silenced MDA-MB-231 cells. Human mammary epithelial cells (HMEC) were used as negative control cells. These data indicate that altering PLD gene expression in low- or high-aggressive breast cancer sets the stage for altering expression of other genes vital to cell chemotaxis and metastasis, the effects of which could be compounded following xenotransplantation into an orthotopic model.

### PLD inhibitors, which target both TAN and TAM in the tumor microenvironment, “spare” innate immune system functionality of non-tumor-associated macrophages (peritoneal Mø)

Lastly, we wanted to investigate if the innate immune cells of the mice were affected by the PLD inhibitor treatments in addition to the herein proven effect on the TAMs and TANs. Using chemoattractant-mediated chemotaxis as a physiological measure of leukocyte function *ex vivo* from peripheral blood polymorphonuclear leukocytes (PMNs,) as well as peritoneal macrophages (MΦ), we found that vehicle-treated PMNs responded positively to fMLP stimulation in SCID mice (Fig 9B). However, chemotaxis was significantly decreased in both the FIPI- and VU0155072-2-treated animals (Fig 9B). Vehicle-treated peritoneal MΦ also responded positively to MCP-1 stimulation (Fig 9D).

Surprisingly, peritoneal MΦ from inhibitor-treated animals were resistant to chemotaxis inhibition (Fig 9D); in fact, a slight activation effect was observed, indicating that these cells were “emboldened” in their physiological response to chemoattractants (Fig 9D). These data suggest the initial inflammatory response of both PMNs and peritoneal MΦ during chemotaxis remained intact in these SCID animals. However, we found that PMNs were targeted to both PLD inhibitors, while peritoneal MΦ were resistant to both of the PLD inhibitors. The chemotaxis data also broadly coincided with PLD enzymatic activity (Fig 9C and 9E).

Data from Fig 9 emphasizes that the innate inflammatory response, as measured by chemotaxis is intact and PLD inhibitors could have a side effect by affecting functionality of peripheral neutrophils without impacting functionality of peritoneal macrophages, which are different from TAMs, the target we have proved to be affected by the inhibitors.

## Discussion

The signaling protein phospholipase D (PLD) is central to cell migration. It is involved in both leukocyte chemotaxis and cancer cell invasion. Using a well-controlled *in vivo* study of human breast cancer xenotransplanted into SCID mice and subsequent molecular biology, enzymology and pathology analyses, the data presented herein provides evidence that PLD2 expression is important to tumor-associated macrophages in human breast cancer growth and cell migration.

We recently showed that the signaling enzyme PLD2 is present in breast tumors, and mice injected with lowly invasive MCF-7 cells overexpressing PLD2 developed tumors more readily, whereas mice injected with highly invasive MDA-MB-231 cells that were stably silenced for PLD2 appeared as negative controls [31]. Since the tumor promoting effects in these immune cells were abolished upon inhibition of the enzyme phospholipase D, this indicates that this PLD from the immune cells, which functioned as part of an inflammatory response/process, also has a key role in breast cancer progression. Overexpressing PLD resulted in larger mammary and axillary tumors, faster tumor onset and more lung metastases [31]. Conversely, silencing the PLD2 gene in cancer cells or implanting mice with micro-osmotic (Alzet) pumps containing the PLD small-molecule inhibitors FIPI and VU0155072-2 resulted in smaller tumors and fewer lung metastases [31].

Data from this present study indicates that although PLD2 gene expression in breast cancer was not affected by small-molecule inhibitor treatment, its protein expression was reduced after such treatment, which also negatively impacted PLD2 lipase activity. Along with tumor-related issues, xenotransplanted mice also experienced early stages of an inflammatory/immune response that coincided with the severity of tumorigenesis. In the context of tumor progression, the loss of immune cells like TAMs or TANs coincided with alteration in tumor growth and hampered the next step, which was metastasis.

To our surprise, we found that the FIPI and VU0155072-2 PLD inhibitors also inhibited leukocyte (TAM and TAN) recruitment to the tumors, which suggests a shift in macrophage polarization occurred from M2 to that of the less invasive and anti-tumoral M1 macrophage in the PLD-specific inhibitor-treated samples. Additionally, the use of PLD inhibitors could be a means to recruit M1 macrophages to the tumor microenvironment, which could yield a more favorable outcome in terms of patient health.

The Ly6G antibody we used for this study for Figs 3 and 5 is the RB6-8C5 clone and has the potential to react with both Ly6G+ and Ly6C+ cells, although the specificity for Ly6C is much weaker. Ly6C+-containing cells include monocytes that are mobilized from the bone marrow and the spleen and recruited to cancer cell-targeted tissues, such as the lungs. The positive reactivity for Ly6G staining in our xenotransplanted lung samples (Fig 5) suggests that a small portion of these cells that we consider to be neutrophilic could in fact be monocytic. Monocytes in SCID mice range from ~4–7%, which is dependent upon gender with females having slightly more monocytes than males [53]. Therefore, the potential exists that up to 7% of the Ly6G staining we detected in xenotransplanted mouse lungs could instead be monocytic Ly6C protein. Further, a recent study has documented preferential accumulation of Ly6G+ neutrophils versus Ly6C+ monocytes in lungs of a mouse model that closely mimics human breast cancer [54], which we feel supports our data that indicates we detected Ly6G+ neutrophils in xenotransplanted SCID mouse lungs well beyond that of Ly6C+ monocytes.

In SCID mice, the acquired immune system (which includes both B- and T-cells) is compromised but not the compartment of innate immune system (which includes the complement and natural killer cells). As the SCID mouse strain is known to be “leaky” as a function of time, resident macrophages of SCID mice could be phenotypically and functionally altered to a M2-like phenotype and that M1 and M2 macrophages could be selectively induced [55–57]. Our data shows a decreased involvement of F4/80-, Arg1- and Ly6G-mediated pathways in our orthotopic breast cancer xenograft model as a result of PLD-specific inhibitor treatment.

## Conclusions

Data from this current study indicates that primary breast tumors increased in both tumor volume and metastases as a function of time and that PLD-specific inhibitors decreased both of these factors, which strongly suggests PLD2 has a role in breast tumor progression. This work and an earlier study [31] demonstrates that PLD inhibitors decrease breast cancer tumor growth and reduce the appearance of TAMs and TANs infiltrated in the tumor microenvironment, indicating a potential therapeutic value for these molecules. For the mechanism, the decrease of TAMs and TANs could be derived from the diminished presence in tumor tissues of cytokines GM-CSF and IL-8, as well as a decreased gene expression of the small GTPase Rac2, key for leukocyte chemotaxis. Another novel finding uncovered in this study is while PLD inhibitors affected the functionality of non-tumor associated neutrophils (peripheral blood PMN), they did not inhibit peritoneal macrophages, indicating that at least some part of the innate immune system is still operative even in the presence of effective tumor-decreasing PLD inhibitors.
